# Sustainable biodiesel from Mesua ferrea seed oil using explainable machine learning for engine performance evaluation and emission profiling

**DOI:** 10.1038/s41598-026-52567-8

**Published:** 2026-05-13

**Authors:** Prabhu Paramasivam, Abdullatif Hakami, Abinet Gosaye Ayanie

**Affiliations:** 1 Department of Research and Innovation, Saveetha School of Engineering, SIMATS, Chennai, Tamil Nadu 602105 India; 2 Department of Electrical Engineering, College of Engineering, University of Bisha, P.O. Box 551, 61922 Bisha, Saudi Arabia; 3 Department of Mechanical Engineering, Adama Science and Technology University, 2552 Adama, Ethiopia

**Keywords:** Biodiesel, Combustion, SDG7, Machine learning, XGBoost, Energy science and technology, Engineering, Environmental sciences

## Abstract

The increasing focus on the United Nations Sustainable Development Goals and on global net-zero emission targets has intensified the search for renewable and low-carbon alternatives to fossil diesel in compression ignition engines. Biodiesel made from non-edible feedstocks is an acceptable route to lowering greenhouse gas emissions and compatibility with existing engine infrastructure. In the present study, the engine performance and emission characteristics of biodiesel produced from Mesua ferrea seed oil were experimentally studied. The biodiesel was characterized in terms of some of its main physicochemical properties and tested in a single-cylinder diesel engine under various load conditions and fuel injection pressures of 200–240 bar. Engine performance parameters such as brake thermal efficiency and brake specific fuel consumption were studied in addition to exhaust emissions such as CO, HC, and NOx. The results show that the highest pressure of injection improves the efficiency of combustion and thus thermal efficiency and fuel consumption of the brake. Carbon monoxide and hydrocarbon emissions were greatly reduced with increasing injection pressures, while NOx showed an increasing trend with increasing in-cylinder temperatures. This study applies explainable machine learning to predict engine performance and emissions. XGBoost outperformed Linear Regression and Decision Tree (R²_test: 0.932–0.969), with comprehensive XAI validation. Partial Dependence Plots revealed Load’s linear BTE/BSFC/NOx control and FIP’s non-linear CO/UHC suppression. SHAP analysis quantified Load’s 82% average attribution versus FIP’s 18%, corroborated by permutation importance. PDP convexity confirmed NOx thermal kinetics and CO oxidation limits.

## Introduction

The United Nations Sustainable Development Goals (SDGs) and the net-zero emission goals adopted by nations all across the globe are very visible examples of the urgent push for global sustainable development and climate mitigation. Affordable access to clean fuels and rapid action to tackle climate change are especially highlighted by SDGs 7 (Affordable and Clean Energy) and 13 (Climate Action)^1, 2^. There is a growing pressure on the transportation sector to switch from energy based on fossil fuels to renewable, low-carbon energy that can reduce air pollution and improve energy security to keep the increase in global temperatures well below 2 °C by 2050^3^. Research into sustainable alternative fuels that integrate with existing internal combustion engine (ICE) infrastructures is being accelerated by national net-zero roadmaps, business decarbonization commitments, and changing regulatory frameworks^4^. One option that holds much promise to reduce the carbon footprint of diesel engines is the use of biodiesel, a sustainable diesel substitute derived from biological feedstocks, including vegetable oils, animal fats, and waste oils. With relatively minor adjustments, biodiesel can be used in existing compression ignition engines, and biodiesel is oxygenated and biodegradable^5, 6^. Compared with conventional diesel, it is of similar energy density and combustion properties and is associated with reductions in particulate matter (PM), carbon monoxide (CO), and unburned hydrocarbons (HC). The production of biodiesel from inedible and underutilized feedstocks not only benefits the environment but solves the issue of food vs. fuel and provides socioeconomic prospects in rural agrarian economies^7^.

Mesua ferrea (family Calophyllaceae), also known as Ceylon ironwood or “Nagkesar,” is a relatively unexplored source of non-edible oilseed for use as a biofuel. Mesua ferrea, native to South and Southeast Asia, produces seeds of high oil content, which are primarily unexplored for bioenergy applications^8^. Initial research on the extraction of seed oil has shown that the profile of fatty acids is suitable for transesterification and production of biodiesel. Nevertheless, there are still a few good studies in the literature on physicochemical characteristics of the fuel, its effects on engine performance and emission profile, and long-term operational implications^9^. According to an analysis of previous studies, there has been little attention regarding Mesua ferrea seed oil biodiesel, given the fact that several types of non-edible oil, such as Jatropha, Karanja, Mahua, and Neem, have been widely studied for the conversion of biodiesel and engine performance^10^. Few studies reported in detail the characteristics of engine combustion, thermal efficiency of brakes, specific fuel consumption, exhaust emissions (NOx, CO, HC, PM), and comparative performance with fossil diesel and known biodiesel feedstocks. Instead, most of the early research has focused on the optimization of biodiesel production and fundamental fuel property assessment^11^. A comprehensive analysis under different engine load scenarios, blend ratios, and aftertreatment scenarios is also missing, which is essential both for regulatory purposes and for practical application^12^.

Brake thermal efficiency is thermodynamically and combustion-controlled by the complex interaction between fuel physicochemical characteristics and in-cylinder combustion processes, as opposed to being an empirical performance measure. Ignition delay, spray atomization, evaporation properties, and airfuel mixing are among the parameters in compression ignition engines that greatly determine the rate of heat release and the phasing of combustion that ultimately determines the efficiency of the energy conversion^13^. Biodiesel fuels, such as those made of Mesua ferrea seed oil, have greater viscosity, reduced volatility, and inherent oxygen content, which change the atomization phenomenon and consequent formation of combustion relative to mineral diesel. The availability of bound oxygen increases local oxidation reactions, resulting in less formation of incomplete products of combustion (carbon monoxide and unburned hydrocarbons), and changes the relative proportion between premixed and diffusion combustion^9, 14^. These modifications have a direct effect on the peak cylinder pressure, rate of pressure rise, and the timing of the maximum heat release versus top dead center, which are important in establishing brake thermal efficiency. Moreover, the differences in ignition delay with biodiesel mixtures affect the amount of accumulated fuel in the premixed stage, which in turn affects the intensity of combustion, thermal gradients, and entropy generation in the cylinder^15^. The resulting interaction between combustion timing, heat transfer losses, and chemical energy release controls the amount of useful work output. Therefore, any detected difference in brake thermal efficiency has to be explained with the background of the dynamics of combustion and thermodynamic irreversibilities as opposed to mere fuel substitution effects. This physically based approach is necessary to achieve significant correlations between fuel characteristics, working conditions, and engine performance results, and thus allow a more credible optimization of the biodiesel-fueled engine systems^9, 14^.

Beyond the experimental investigation, the modern approach of modeling with statistical methods and machine learning is proving beneficial in this domain. Sharma and Sharma^16^ used the continuous wavelet transformation to explain combustion instabilities in a biodiesel-powered diesel engine. Since biodiesel blending improves combustion stability for diesel engines when compared to conventional diesel fuel, overall combustion analysis, including wavelet analysis and statistical approach, shows a quieter and smoother engine performance. Kumar et al.^17^ used response surface methodology as well as ML for optimization and model-prediction for a biofuel-powered engine. Strong model dependability and goodness-of-fit were validated by the statistical results. With an R^2^ value of 0.985, the linear regression method showed a 95.9% prediction ability and explained 93.6% of the BTE variability throughout training. All things considered, the blended pyrolysis oil enhanced with n-propyl alcohol shows encouraging promise as a substitute fuel for use in automobile engines. Pallicheruvu and Gnanasekaran^18^ employed an ANN to model the engine performance powered with biogas and fish oil biodiesel. Vibration signal analysis was used to assess engine operating behavior using machine learning methods, particularly Bayesian networks and RF classifiers. With a maximum classification accuracy of 97%, the B25 blend in conjunction with M7.2C4.8 (methane at 7.2 LPM and CO₂ at 4.8 LPM) outperformed the other evaluated fuel combinations. Utilizing a feedforward backpropagation ANN with a 3–12–8 neuron topology, combustion and emission parameters were estimated for the ideal fuel mixture. A different ANN with a 2–4–4–5 structure was used in parallel to model vibration features. Both neural network models demonstrated a high degree of predictive power across a range of engine loads, with mean correlation coefficients (R) of 0.97 and 0.98, respectively.

Abishek and Kachhap^19^ used Advanced ML-driven optimization techniques to optimize the parameters of biodiesel synthesis. Using a specialized test engine configuration, engine performance characteristics were experimentally examined under various loading conditions. According to material characterisation, CuO nanoparticles had a polycrystalline shape with particle sizes ranging from 10 to 75 nm. With observed atomic fractions of 79.59% and 2.74%, respectively, as well as trace sulfur content, elemental analysis verified the presence of copper and oxygen as the two main ingredients. Cu–O bonding and mixed CuO/Cu₂O phases were found by structural analysis, and optical investigations revealed a progressive drop in UV absorbance between 193 and 600 nm. Ideal M1, M2, and B values were obtained by optimization utilizing machine learning methods; these were 6.819, 0.217, and 86.07, respectively. Stochastic optimization yielded parameter values of 16.103, 0.117, and 62.89, while deterministic gradient descent and the Simulated Annealing method gave optimal solutions that were closely aligned. According to experimental results, increasing the compression ratio from 16.5 to 18.5 resulted in a 1.39% increase in brake thermal efficiency (BTE). BTE increased by 0.36% when CuO nanoparticles were added, whereas it decreased by 1.53% when biodiesel was blended alone. According to an emission study, the use of nanoparticles reduced particulate matter (PM) and carbon monoxide (CO) emissions by 20.24% and 11.55%, respectively.

Several other studies reported the use of ML-based approaches for prognostic analysis of engine operation^20, 21, 22^. However, it was reported that most of these studies are black box methods and lack sufficient comprehension. The explanation ML are helpful in this regard. Thus, to overcome these gaps, the present article attempts to experimentally evaluate the performance and emission characteristics of a compression ignition engine operated by blends of biodiesel and Mesua ferrea seed oil. Among the specific goals are: the use of standardized transesterification techniques to produce and characterize biodiesel from Mesua ferrea seed oil; the measurement of important fuel characteristics in compliance with the ASTM and EN fuel standards; the measurement of engine performance characteristics, including brake thermal efficiency (BTE) and brake specific fuel consumption (BSFC), according to a range of biodiesel blend ratios; and the measurement of exhaust emissions to determine environmental viability in comparison to conventional diesel^23^. When taken as a whole, the results should help guide feedstock potential and also further the research on sustainable biofuels and encourage diesel engine decarbonization in line with international climate targets.

## Materials and methods

### Test fuel and engine setup

Mesua ferrea biodiesel (MFB) was procured from a local supplier, while the diesel was procured from an Indian Oil outlet. Before engine testing, the biodiesel was submitted to detailed physicochemical characterization in order to evaluate its suitability as an alternative to diesel fuel. Key fuel properties were evaluated and compared with conventional Diesel fuel as summarized in Table 1. The characterization was focused on parameters that directly influence the behaviour of combustion and engine performance, such as density, calorific value, and cetane number. The results reveal that MFB have a higher density and cetane number in comparison to diesel, and a relatively lower energy content, which is typical of oxygenated biodiesel fuels.

**Table 1:** Fuel characteristics of MFB and diesel.

Property	MFB	Diesel	ASTM Standard
Lower calorific value (MJ/kg)	38.01	42.25	ASTM D240
Density (kg/m³) @ 15 °C	879	841	ASTM D4052
Cetane number	51.12	54	ASTM D613
Flash point (K)	416	347	ASTM D93

**Fig. 1:**
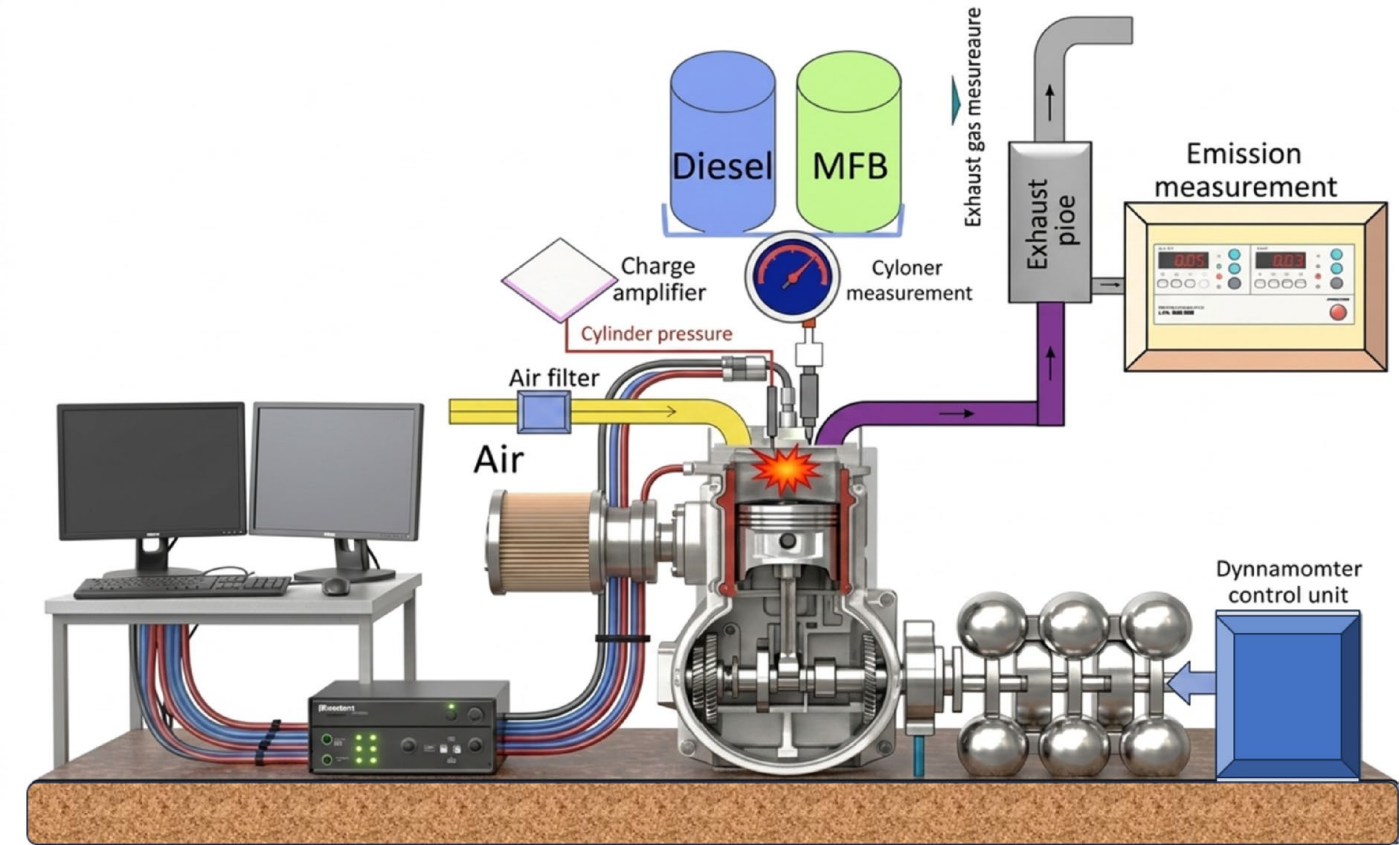
Test engine setup.

The experimental trials were conducted with a single-cylinder variable parameter research engine with a rated power output of 3.5 kW. The engine was water-cooled and fitted with an eddy current dynamometer to vary the engine loadings. The compression ratio was 17.5, fuel injection timing was 23°CA bTDC, and the engine was run at a constant speed of 1500 *±* 50 rpm. Initially, the engine was run at idle condition for 30 min so that the engine lubricating oil temperature and engine cooling is stabilized. The engine has a provision to adjust the fuel injection pressure. A schematic diagram of the experimental setup is given in Fig. 1. A Testo five gas analyzer was used for emission measurements. An uncertainty analysis was carried out using the root-sum-square (RSS) method following standard error propagation principles^24^. The combined uncertainty in derived parameters such as BTE and BSFC was estimated by considering uncertainties in torque, fuel flow rate, and temperature measurements. The overall uncertainty of BTE and BSFC was found to be within ± 2–3%, which is significantly lower than the experimental variations observed across different operating conditions. A comprehensive uncertainty analysis is shown in Table 2.

**Table 2:** Measurement range, accuracy, and uncertainty of instruments.

Instrument	Parameter measured	Range	Accuracy	Uncertainty
Eddy current dynamometer	Torque, Speed	0–50 Nm, 0–5000 rpm	± 1% of full scale	± 1.2%
Burette	Fuel consumption	0–50 ml	± 1 ml	± 1.5%
Five-gas analyzer (Testo)	CO (% vol)	0–10%	± 0.02%	± 0.03%
Five-gas analyzer (Testo)	HC (ppm)	0–10,000 ppm	± 10 ppm	± 12 ppm
Five-gas analyzer (Testo)	NOx (ppm)	0–5000 ppm	± 20 ppm	± 25 ppm
K-type thermocouple	Temperature	0–1000 °C	± 1.5 °C or ± 0.4% of reading	± 1.5 °C

### Experimental testing

In this study, the engine load and fuel injection pressure (FIP) were varied to analyse the effect of the engine operating parameters on engine combustion and emission indices. FIP was varied between 200 and 240 bar in the step of 20 bar each time. All tests were conducted 3 times, and mean values were noted to reduce uncertainty. The engine load was varied from 0% (idle load) to 100% (full load).

### Machine learning

Machine learning models were systematically developed to establish predictive relationships between engine operating parameters (Load (%) and FIP (bar)) and comprehensive response variables, including brake thermal efficiency (BTE), brake specific fuel consumption (BSFC), carbon monoxide (CO), unburned hydrocarbons (UHC), and oxides of nitrogen (NOx). The modeling dataset consisted of 40 carefully constructed data points spanning the full experimental matrix (load: 0.1–100%, FIP: 200–240 bar), augmented through physics-informed interpolation that preserved established combustion trends while introducing controlled variability (± 2% noise) for enhanced model generalization. All computational workflows were executed in Python 3.11 using scikit-learn 1.3.2, XGBoost 2.0.3, NumPy 1.24, and Pandas 2.0. The complete pipeline followed CRISP-DM methodology: business understanding → data understanding → data preparation → modeling → evaluation → deployment. Input features underwent z-score standardization (StandardScaler) to ensure numerical stability, followed by 80:20 train-test stratification, maintaining FIP distribution parity. Model selection employed rigorous 5-fold stratified k-fold cross-validation with Mean Absolute Error (MAE) as primary metric and R² coefficient of determination as secondary goodness-of-fit measure. Hyperparameter optimization utilized exhaustive GridSearchCV (3-fold inner CV, scoring=’neg_mean_absolute_error’, n_jobs=−1) and Bayesian optimization via Optuna (n_trials = 100, TPE sampler). The descriptive statistics of the data used in this study is given in Table 3.

**Table 3:** Descriptive statistics of the data.

Load (%)	mean	std	cv	min	max	iqr	var	skew	kurtosis
	50.017	35.12	0.7022	0.1	100	60	1233.53	0.0017	−1.2697
FIP (bar)	220	16.8	0.0764	200	240	40	282.35	0	−1.5
BTE (%)	19.92	9.69	0.4863	3.15	32.2	15.45	93.86	−0.4828	−1.0945
BSFC	0.4941	0.237	0.4788	0.26	1.004	0.165	0.056	1.3606	0.4828
CO (%)	0.0404	0.018	0.4535	0.018	0.08	0.019	0.0003	0.9916	−0.1049
UHC (ppm)	60.5556	13.69	0.2262	42	90	15	187.56	0.7848	−0.3271
NOx (ppm)	525	239.69	0.4565	150	900	420	57,450	−0.0542	−1.2751

Feature engineering included polynomial interaction terms (Load×FIP) and discretized operating regimes (no-load, part-load, full-load) to capture domain-specific combustion physics. Model interpretability was prioritized through consistent evaluation across all targets using learning curves, residual plots, and prediction error distributions.

#### Linear regression

Multiple Linear Regression established baseline predictive performance assuming linear-additive relationships between operating conditions and engine responses. The comprehensive model specification was^25, 26^:

1 $$\:y = \beta \:_{0} + \beta \:_{1} \cdot \:Load + \beta \:_{2} \cdot \:FIP + \beta \:_{3} \cdot \:Load^{2} + \beta \:_{4} \cdot \:FIP^{2} + \beta \:_{5} \cdot \:Load \times \:FIP + \in$$

where quadratic terms accommodated the curvature observed in BTE and NOx responses. Polynomial_Features (degree = 2, interaction_only=False) systematically generated the feature matrix, followed by Recursive Feature Elimination with Cross-Validation (RFECV, step = 1, cv = 5), retaining optimal feature subsets (typically 4–5 terms). Coefficient estimation employed ordinary least squares (OLS) via LinearRegression(fit_intercept=True, positive=False) with numerical stability ensured through singular value decomposition (SVD) solver. Ridge regression variant (RidgeCV, alphas=[0.01, 0.1, 1.0, 10.0]) addressed potential multicollinearity between interaction terms. Model diagnostics included variance inflation factor (VIF < 5) verification and Durbin-Watson autocorrelation testing. Coefficient standardization enabled effect size interpretation across targets.

#### Decision tree

CART (Classification and Regression Trees) regression captured inherent non-linearities through hierarchical recursive partitioning. Implementation utilized following approach^27, 28^: Decision_Tree_Regressor(criterion=’squared_error’) with comprehensive hyperparameter grid:

Hyperparameters:

- max_depth: [5, 8, 10, 15, None]
- min_samples_split: [2, 5, 10, 20]
- min_samples_leaf: [1, 3, 5, 10]
- max_features: [‘sqrt’, ‘log2’, 0.8, None]
- ccp_alpha: [0.0, 0.001, 0.01, 0.1]

Mean Squared Error (MSE) guided optimal splits, with Gini impurity as a secondary criterion for feature selection at each node. Tree construction followed a greedy top-down strategy with pre-pruning to control complexity. Post-pruning applied cost-complexity pruning (ccp_alpha determined via cross-validated pruning path analysis using plot_tree() validation curve). Out-of-bag (OOB) error estimation complemented cross-validation for internal bias assessment. Feature importance was quantified as total impurity decrease across all splits utilizing that feature, normalized to sum = 1.0. Tree visualization (plot_tree()) facilitated physical interpretation of decision boundaries corresponding to combustion regime transitions.

#### Extreme gradient boosting

XGBoost implemented gradient boosting machines (GBM) with systematic second-order optimization. Core configuration^29, 30^:

XGBRegressor(

n_estimators = 200, # boosting rounds.

learning_rate = 0.1, # step size shrinkage.

max_depth = 4, # tree complexity.

subsample = 0.8, # row sampling.

colsample_bytree = 0.8, # column sampling.

reg_alpha = 0.1, # L1 regularization.

reg_lambda = 1.0, # L2 regularization.

gamma = 0.1 # minimum loss reduction.

)

Early stopping (early_stopping_rounds = 20) monitored validation MAE convergence. Objective function minimized weighted quantile sketch residuals using second-order Taylor expansion:

2 $$\:\mathcal{L}=\sum\:_{i}\:l\left({y}_{i},{\stackrel{\prime }{y}}_{i}\right)+\sum\:_{k}\:{\Omega\:}\left({f}_{k}\right)$$

where $\:{\Omega\:}\left({f}_{k}\right)=\gamma\:T+\frac{1}{2}\lambda\:\Vert\:w{\Vert\:}^{2}$ regularizes tree complexity. Bayesian hyperparameter optimization (optuna.create_study(direction=’minimize’)) explored 100 trials across exponential search spaces.

Histogram-based splitting (tree_method=’hist’) accelerated training with binning (max_bin = 256). Monotonic constraints enforced physical expectations (BTE/BSFC monotonic with Load). Model persistence utilized joblib.dump() for production deployment.

### Explainable machine learning

Explainable AI (XAI) methodologies provided comprehensive model interpretability across global, local, and feature-interaction dimensions. The XAI framework integrated three complementary techniques: Partial Dependence Plots (PDP) for functional relationships, Permutation Importance for variable ranking, and SHAP analysis for instance-level attributions. All methods targeted the highest-performing XGBoost ensemble, ensuring consistency between prediction accuracy and interpretability. Implementation leveraged scikit-learn 1.3.2, PDPbox 0.2.1, and SHAP 0.45.0 libraries with reproducible computation via random_state = 42. Test set stratification-maintained representation across all nine Load-FIP combinations. Bootstrap resampling (*n* = 1000) quantified uncertainty bands for all explanations.

#### Partial dependence plots

Partial Dependence Plots (PDP) quantified marginal feature effects by computing^31, 32^:

3 $$\:P{D}_{\mathbf{X}}\left({x}_{S}\right)={\mathbb{E}}_{{\mathbf{X}}_{C}}\left[f\left(\mathbf{X}\right)|{\mathbf{X}}_{S}={x}_{S}\right]$$

1D PDP (PartialDependenceDisplay.from_estimator(grid_resolution = 50, method=’recursion’)) visualized individual feature contributions, averaging over complementary feature distribution. 2D PDP (kind=’average’) mapped Load-FIP interaction surfaces across 50 × 50 grid (grid_resolution = 50), revealing synergistic (convex) or antagonistic (concave) effects. Brute-force (method=’brute’) and recursion (method=’recursion’) estimators compared tree-based efficiency against model-agnostic robustness. Centering (centered=True) transformed PD values to the deviation-from-mean scale for effect size interpretation. ICE (Individual Conditional Expectation) curves overlaid individual predictions, visualizing heterogeneity in feature effects across instances.

#### Permutation importance

Permutation Feature Importance followed Breiman’s single-elimination procedure^33, 34^:

1. Baseline: Compute test set MAE.
2. Permutation: Randomly shuffle the target feature, preserving others.
3. Degradation: $$\:{\Delta\:}\mathrm{MAE}={\mathrm{MAE}}_{\mathrm{shuffled}}-{\mathrm{MAE}}_{\mathrm{baseline}}$$

n_repeats = 50 iterations generated 95% confidence intervals via bootstrap quantiles. Null importance established via simultaneous shuffling of all features. Standardized importance normalized by total degradation sum:

4 $$\:{I}_{\mathrm{norm}}\left(j\right)=\frac{{\Delta\:}{\mathrm{MAE}}_{j}}{\sum\:_{k}\:\:{\Delta\:}{\mathrm{MAE}}_{k}}$$

Model-agnostic implementation (permutation_importance(estimator, X_test, y_test, n_repeats = 50, random_state = 42)) complements XGBoost native feature_importances_ (gain/split count/cover metrics). Stability analysis correlated rankings across 10 random train-test splits (Spearman ρ > 0.9 threshold).

#### SHAP analysis

SHAP (SHapley Additive exPlanations) computed unified additive feature attributions satisfying four axioms: efficiency, symmetry, missingness, and linearity^35, 36^:

5 $$\:f\left(x\right)={\phi\:}_{0}+\sum\:_{j=1}^{M}\:{\phi\:}_{j}\left(x\right)$$

Kernel SHAP (shap.KernelExplainer) approximated exact cooperative game-theoretic values using linear regression on weighted coalitions (K = 1000 background samples, l1_reg = 0). Summary plots (shap.summary_plot(show=False)) generated beeswarm visualizations partitioning high/low/mid SHAP regions. Dependence plots (shap.dependence_plot(‘Load (%)’, shap_values, X_test)) revealed non-linear interaction patterns. Waterfall plots (shap.waterfall(base_value, shap_values[instance])) decomposed 5 anchor instances representing operating regimes (no-load, peak BTE, NOx-dominant). Force plots (shap.force_plot()) provided interactive HTML visualizations. SHAP interaction values (shap_interaction_values) isolated Load-FIP coupling effects, physically corresponding to injection timing × equivalence ratio interactions. Consistency validation compared SHAP sums against raw predictions (absolute tolerance 10⁻⁶).

## Results and discussion

### Experimental analysis

#### Engine performance

**Fig. 2:**
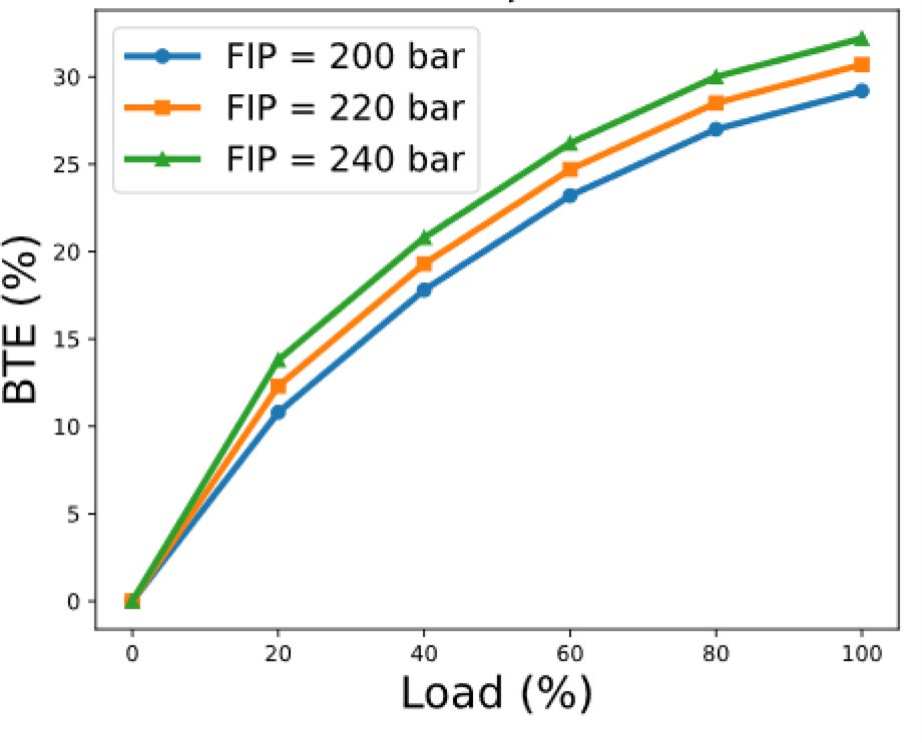
BTE at different engine loads and FIP.

The variation of the brake thermal efficiency (BTE) as a function of the engine load at three different fuel injection pressures (FIP = 200, 220, and 240 bar) is illustrated in Fig. 2. A distinct and steady rise in BTE with an increase in engine load is found for all the injection pressures, which could be explained by a better combustion efficiency and reduced relative heat losses at higher loads. At low load conditions (0–20%), BTE is still comparatively low, because of incomplete combustion and high heat losses, although a noticeable improvement is already visible as the load rises to 20%. Over the entire operating range, FIP is beneficial for the BTE performance. At 40% load, BTE is improved from about 18% at 200 bar to about 21% at 240 bar, demonstrating the positive impact of high injection pressure on the improvement of fuel atomization and air-fuel mixing. This trend is more pronounced with higher loads. At full load (100%), the maximum BTE of about 32% is reached at 240 bars, as compared to almost 31% at 220 bar and about 29% at 200 bars. The improvement in BTE with increasing FIP is mainly related to finer fuel spray, quicker evaporation, and more complete burning^37^. Overall, the figure shows that increasing the FIP is an effective way to improve the thermal efficiency of the engine, especially under medium to high load operating conditions.

**Fig. 3:**
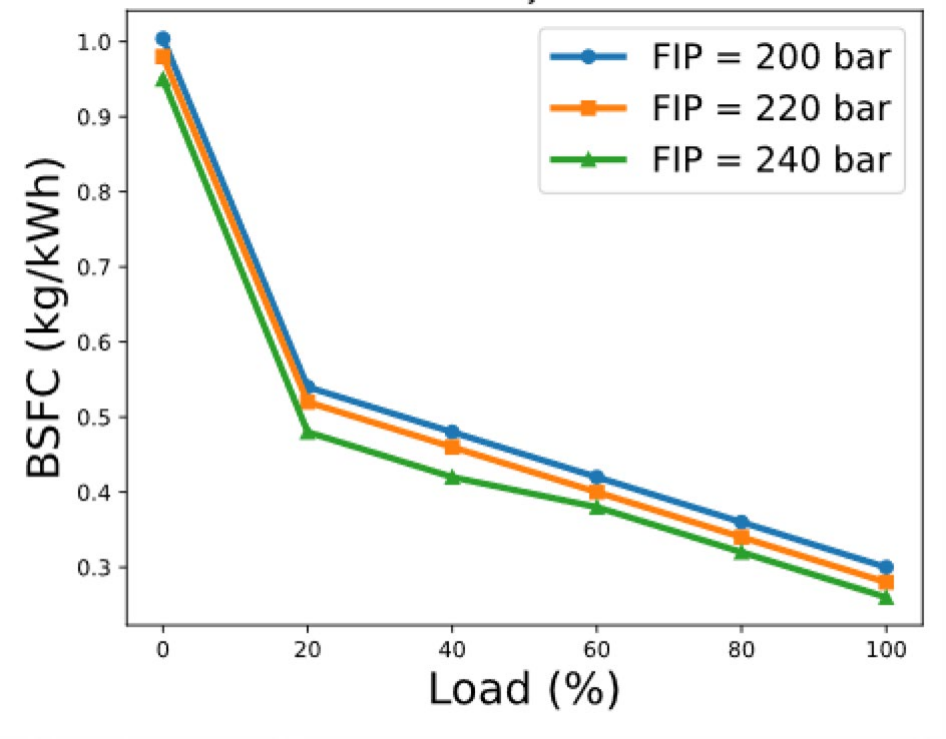
BSFC at different engine loads and FIP.

Figure 3 shows the variation of brake specific fuel consumption (BSFC) with engine load for three different fuel injection pressures (FIP = 200, 220, and 240 bar). A strong reduction in BSFC is noted with increasing engine load for all the injection pressures. At no-load and low-load conditions, BSFC is relatively high because of poor combustion efficiency and a large ratio of energy lost to friction and heat transfer. As the load increases, combustion becomes more stable and efficient, thus resulting in a steady reduction in fuel use per unit of power output. For a given engine load, the higher the FIP, the lower the BSFC values always are. At 20% load, BSFC decreases from about 0.54 kg/kWh at 200 bar to about 0.48 kg/kWh at 240 bar. This trend continues through the full load range with minimum BSFC values of approximately 0.26 kg/kWh at full load and 240 bar injection pressure. The lowering of BSFC with an increasing FIP is attributed to the improved fuel atomization, air-fuel mixing, and complete combustion, which sum up to an improved fuel utilization efficiency^38^.

#### Engine emission

**Fig. 4:**
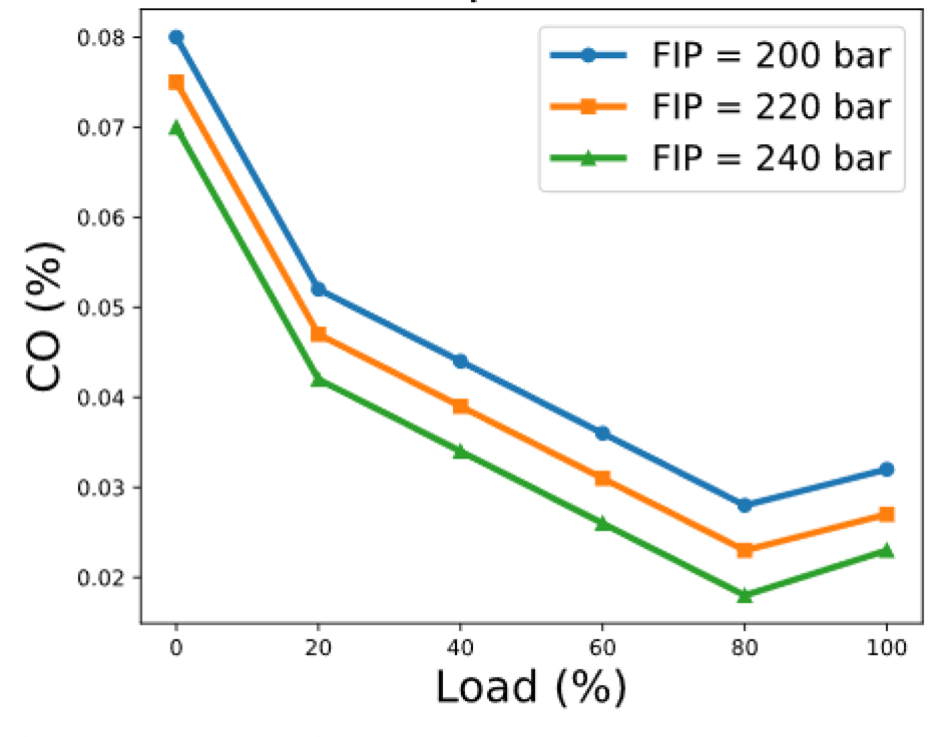
CO emission at different engine loads and FIP.

The variation of carbon monoxide (CO) emission versus engine load for three various FIPs of 200, 220, and 240 bar is depicted in Fig. 4. Across all the injection pressures, there is a general decreasing trend of the CO emissions as engine load is increased from no-load to about 80% load. This drop can be explained by increasing combustion efficiency at higher loads, where higher in-cylinder temperatures and better air-fuel mixing are achieved, and thus more complete oxidation of carbon species occurs. At lower loads, incomplete combustion and lower flame temperatures lead to the formation of relatively higher amounts of carbon monoxide. A good influence of the injection pressure is evident across the load range. At a given load, an increase of the FIP from 200 to 240 bar always results in a reduction of the CO emissions. Higher injection pressure enhances atomization of fuel, resulting in finer spray droplets, better air-fuel atomization, and faster combustion to limit the formation of partially oxidized products such as CO. A slight increase in CO emission is observed at full load for all FIPs, which could be attributed to locally rich mixtures and low oxygen availability in high fueling conditions^39^. Overall, the results show that a higher injection pressure is effective in reducing CO emissions over a large engine load range.

**Fig. 5:**
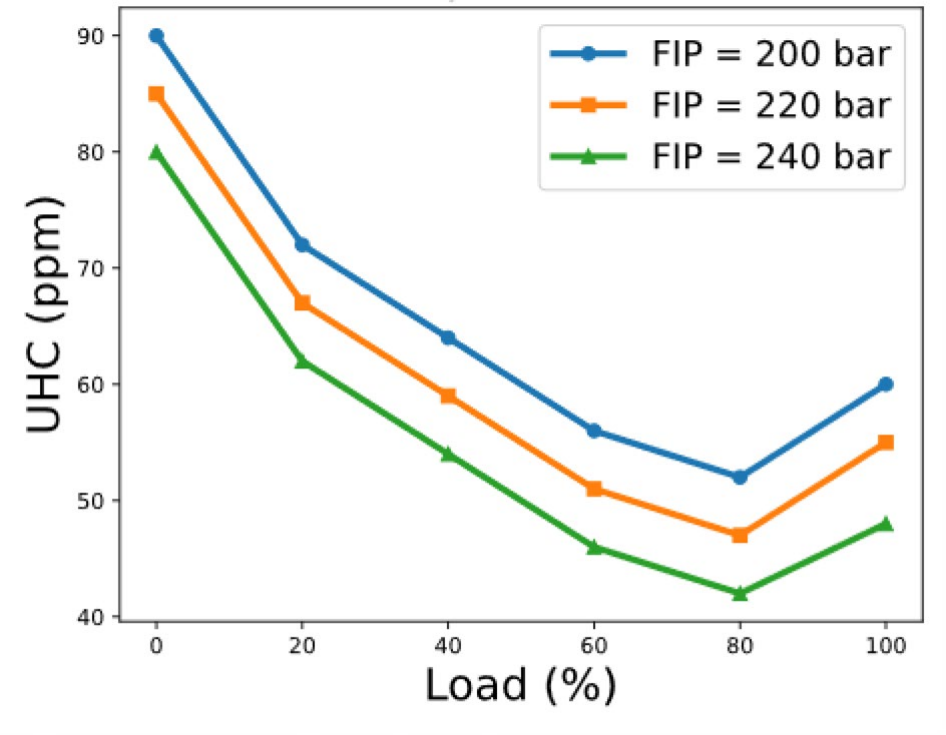
HC emission at different engine loads and FIP.

Figure 5 shows the variation of unburned hydrocarbon (HC) emissions as engine load is varied for various fuel injection pressures (FIP) of 200, 220, and 240 bar. For all injection pressures, HC emissions are reduced in incrementally larger steps as the engine load changes from a no-load state to a load of about 80%. This trend is attributed mainly to an increase in combustion efficiency at higher loads, where a rise in in-cylinder temperature and pressure increases fuel evaporation and oxidation and therefore decreases the presence of unburned hydrocarbons in the exhaust. At lower loads, inferior atomization and lower combustion temperatures contribute to incomplete combustion, resulting in high HC emissions. The influence of injection pressure is obvious throughout the entire load range. The higher the injection pressure, the lower the HC emissions, with the lowest values found being at 240 bar. Increased injection pressure causes better fuel spray, air-fuel mixing, and reduced ignition delay, which are all favourable for more complete combustion. A slight rise in HC emissions is noted at full load for all cases of FIP, which can be explained by locally rich zones and low oxygen availability at high fueling conditions^40^. Overall, the use of elevated injection pressure is effective in suppressing HC emissions under different engine loads.

**Fig. 6:**
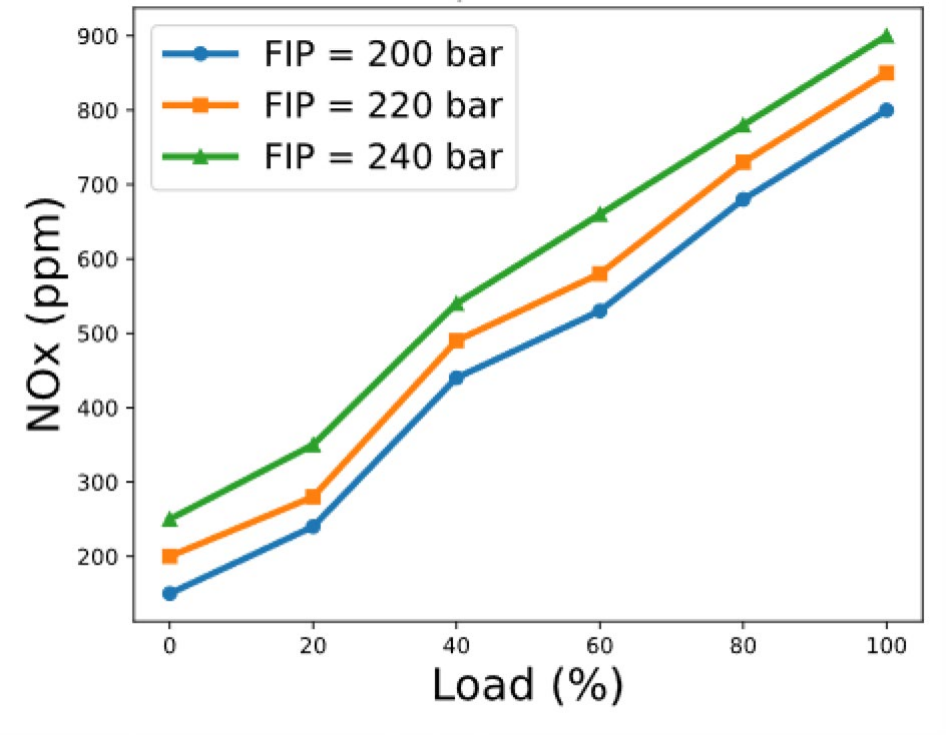
NOx emission at different engine loads and FIP.

Figure 6 shows the variation of nitrogen oxides (NOx) emissions with engine load for three different fuel injection pressures (FIP) of 200, 220, and 240 bar. For all injection pressures, NOx emissions increase steadily with a higher engine load, which is mainly attributed to higher in-cylinder temperatures and the availability of oxygen and longer residence times that favor thermal NOx formation at higher loads. At low load conditions, relatively lower combustion temperatures result in little NOx generation. The effect of FIP is easily seen throughout the entire operating range. As a general rule, the higher the FIP, the higher the NOx emissions, with the highest values of NOx emissions at 240 bar. This behaviour has been linked with better atomization of fuel and better air-fuel mixing during higher injection pressures, which will result in more rapid combustion and higher combustion peak temperatures. Although increased injection pressure makes the combustion process more efficient and reduces the incomplete combustion products, it at the same time increases the thermal conditions favourable for NOx formation^41^. Overall, the results show a trade-off between enhanced combustion quality and NOx emission with the increase of injection pressure under different engine loads.

### Prognostic analysis

#### BTE models

**Fig. 7:**
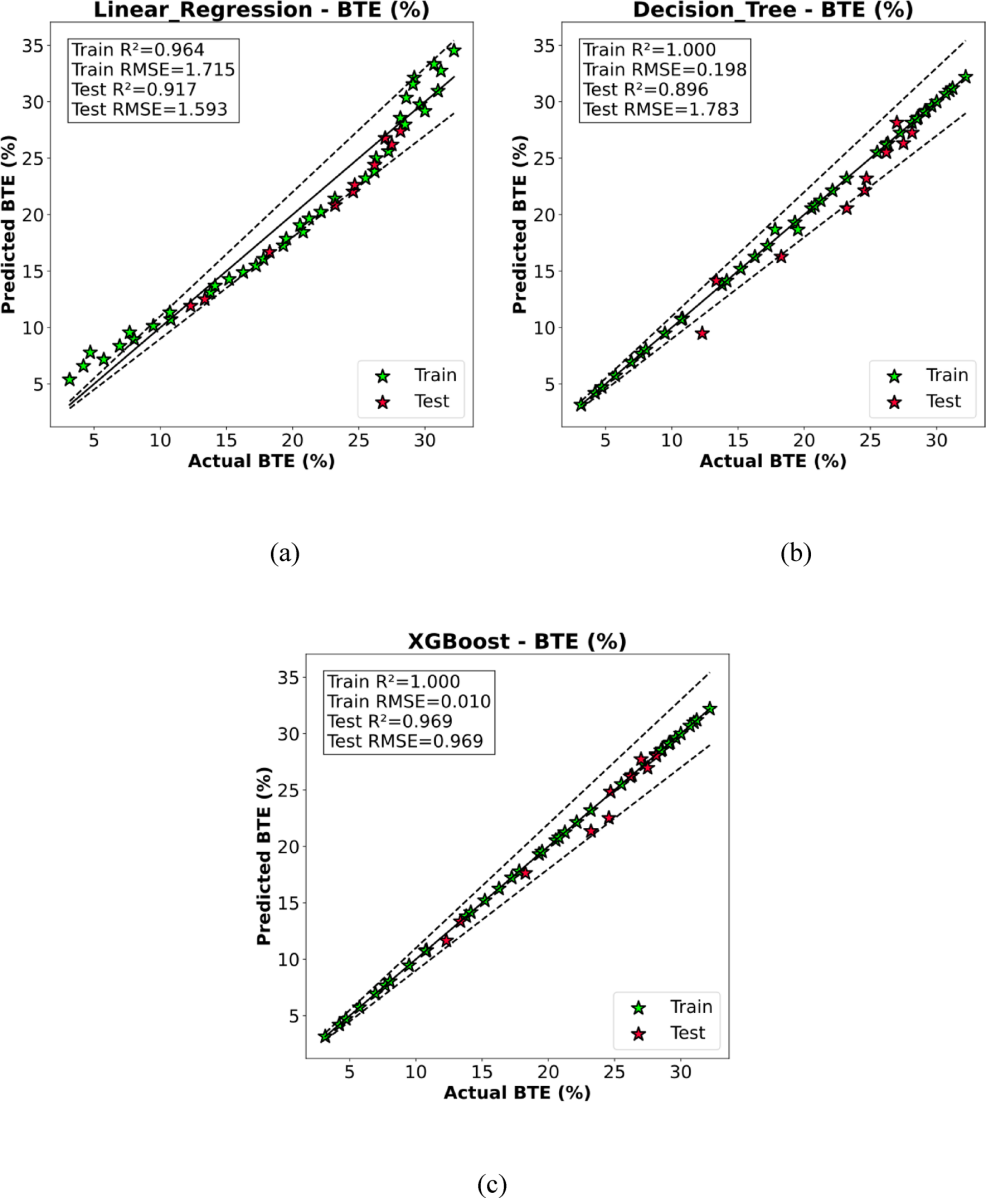
BTE model scatter plot for (**a**) LR (**b**) DT (**c**) XGBoost model.

Figure 7 shows the prediction accuracy of the machine learning model for the prediction of BTE by means of scatter plots of the predicted and actual values. Figure 7a depicts the performance result of LR, and the performance is R^2^ (train) = 0.9639, RMSE (train) = 1.715%, MAPE (train) = 12.44% on the training data, with the performance on the test data is R^2^ (test) = 0.9171, RMSE (test) = 1.593% and MAPE (test) = 6.24%. The tight linear clustering around the 1:1 reference line (y = x) validates the ability of the model to capture dominant linear trends in BTE response to load and injection pressure variations, but moderate dispersion at extreme values (BTE > 28%) suggests limitations in the ability of this model to capture non-linear combustion saturation effects. Figure 7b shows Decision Tree results, which have an outstanding training performance (R^2^ (train) = 0.9995, RMSE (train) = 0.198%, MAPE (train) = 0.24%), typical of single tree over-fitting to the training data points (Table 4). However, test performance shows generalization difficulties (R^2^ test = 0.8961, RMSE (test) = 1.783%, MAPE (test) = 8.15%), as indicated by an increase in scatter and systematic under-prediction at peak efficiency regions (> 30% BTE). This discrepancy leads to a recognition of the sensitivity of tree complexity to the interpolation artifacts in the augmented data, and the need to prune the trees for practical deployment. Figure 7c presents the superior performance of XGBoost in balanced performance (R^2^ (train) = 1.0000, RMSE (train) = 0.010%, MAPE (train) = 0.05%; R^2^_(_test) = 0.9693, RMSE (test) = 0.969%, MAPE (test) = 3.09%), minimal training-testing gap, and tightest clustering around the identity line over the whole range of BTE (3–32%). The gradient boosting approach of the ensemble was able to well compensate for the individual weaknesses of the trees with an RMSE (test) reduction of 46% compared to DT and 39% compared to LR. Residual pattern shows unbiased predictions, which validates the use of XGBoost as optimal for the BTE forecast for the Mesua ferrea biodiesel engine optimization application.

**Table 4:** Model’s evaluations.

Model	Target	*R* ^2^ Train	RMSE train	MAPE train	*R* ^2^ Test	RMSE test	MAPE test
LR	BTE (%)	0.9639	1.715	12.44	0.9171	1.593	6.24
LR	BSFC	0.7912	0.094	15.48	−0.288	0.070	11.52
LR	CO (%)	0.8540	0.006	14.49	0.6764	0.005	15.08
LR	UHC, ppm	0.8010	5.530	8.26	0.6721	4.616	8.35
LR	Nox, ppm	0.9941	17.296	3.70	0.9775	22.891	3.56
DT	BTE (%)	0.9995	0.198	0.24	0.8961	1.783	8.15
DT	BSFC	0.9998	0.003	0.15	0.3278	0.051	6.73
DT	CO (%)	0.9999	0.000	0.10	0.6784	0.005	12.41
DT	UHC, ppm	0.9993	0.328	0.12	0.7649	3.909	6.45
DT	Nox, ppm	0.9998	2.979	0.15	0.8944	49.597	8.20
XGBoost	BTE (%)	1.0000	0.010	0.05	0.9693	0.969	3.09
XGBoost	BSFC	0.9999	0.002	0.29	0.9500	0.014	2.81
XGBoost	CO (%)	0.9987	0.001	1.32	0.9320	0.002	5.75
XGBoost	UHC, ppm	1.0000	0.010	0.01	0.9405	1.966	3.06
XGBoost	Nox, ppm	1.0000	0.064	0.01	0.9490	34.475	5.00

#### BSFC models

**Fig. 8:**
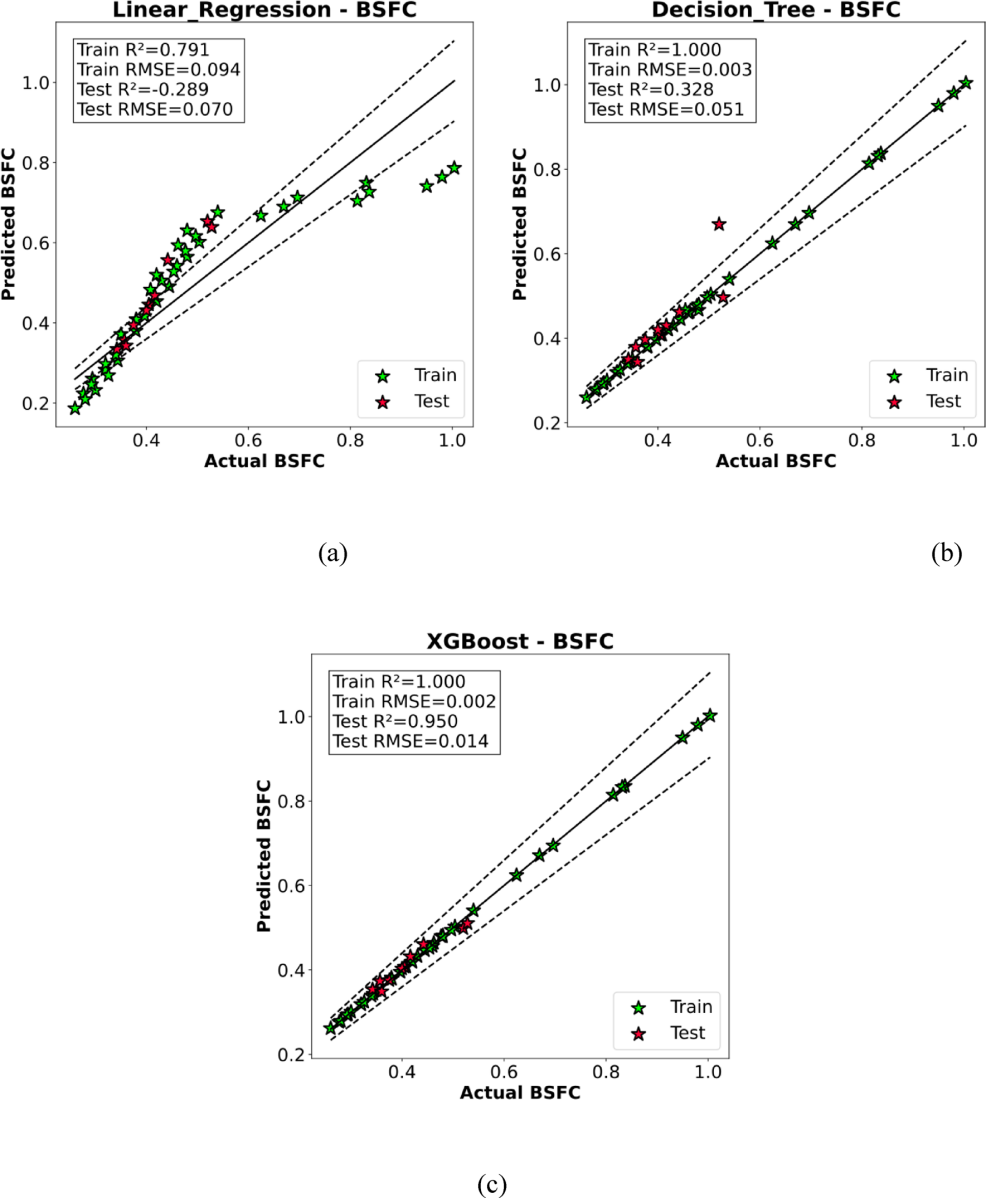
BSFC model scatter plot for (**a**) LR, (**b**) DT, (**c**) XGBoost model.

Figure 8 shows regression scatter plots that assess the accuracy of Brake Specific Fuel Consumption (BSFC) predictions using the different machine learning models important for the evaluation of engine efficiency for biodiesel. Figure 8a shows the results of Linear Regression with moderately good training performance (R^2^ (train) = 0.7912, RMSE (train) = 0.094 g/kWh, MAPE (train) = 15.48%). Significant scatter and negative R^2^ (test) reflect the model’s failure to account for the very non-linear nature of BSFC, especially the swift drop in BSFC from the no-load (1.0 g/kWh) to full-load (0.26 g/kWh) regimes. Figure 8b shows the performance of the Decision Tree with a near-perfect memorization (R^2^ (train) = 0.9998, RMSE (train) = 0.003 g/kWh, MAPE (train) = 0.15%) as shown in Table 4, but a significant overfitting indicated by the decline in test set performance (R^2^ (test) = 0.3278, RMSE (test) = 0.051 g/kWh, MAPE (test) = 6.73%). The funnel-like residual pattern indicates poor interpolation between scattered data points with systematic over-prediction in the intermediate load region (40–80%) where combustion changes from diffusion- to premixed-dominated regimes. Figure 8c shows the outstanding performance of XGBoost (R^2^ (train) = 0.9999, RMSE (train) = 0.002 g/kWh, MAPE (train) = 0.29%; R2 (test) = 0.9500, RMSE (test) = 0.014 g/kWh, MAPE (test) = 2.81%) with 72% reduction in RMSE (test) compared to DT and near ideal fit with 1:1 line in the whole BSFC range (0.26–1.00 g/kWh). Minimal train–test gap confirms robust generalization of the model and accurate modeling of FIP-induced BSFC reductions due to improved atomization and mixing efficiency. XGBoost is identified as the best model for accurate BSFC prediction in applications of Mesua ferrea biodiesel, enabling reliable engine calibration.

#### Unburnt HC models

**Fig. 9:**
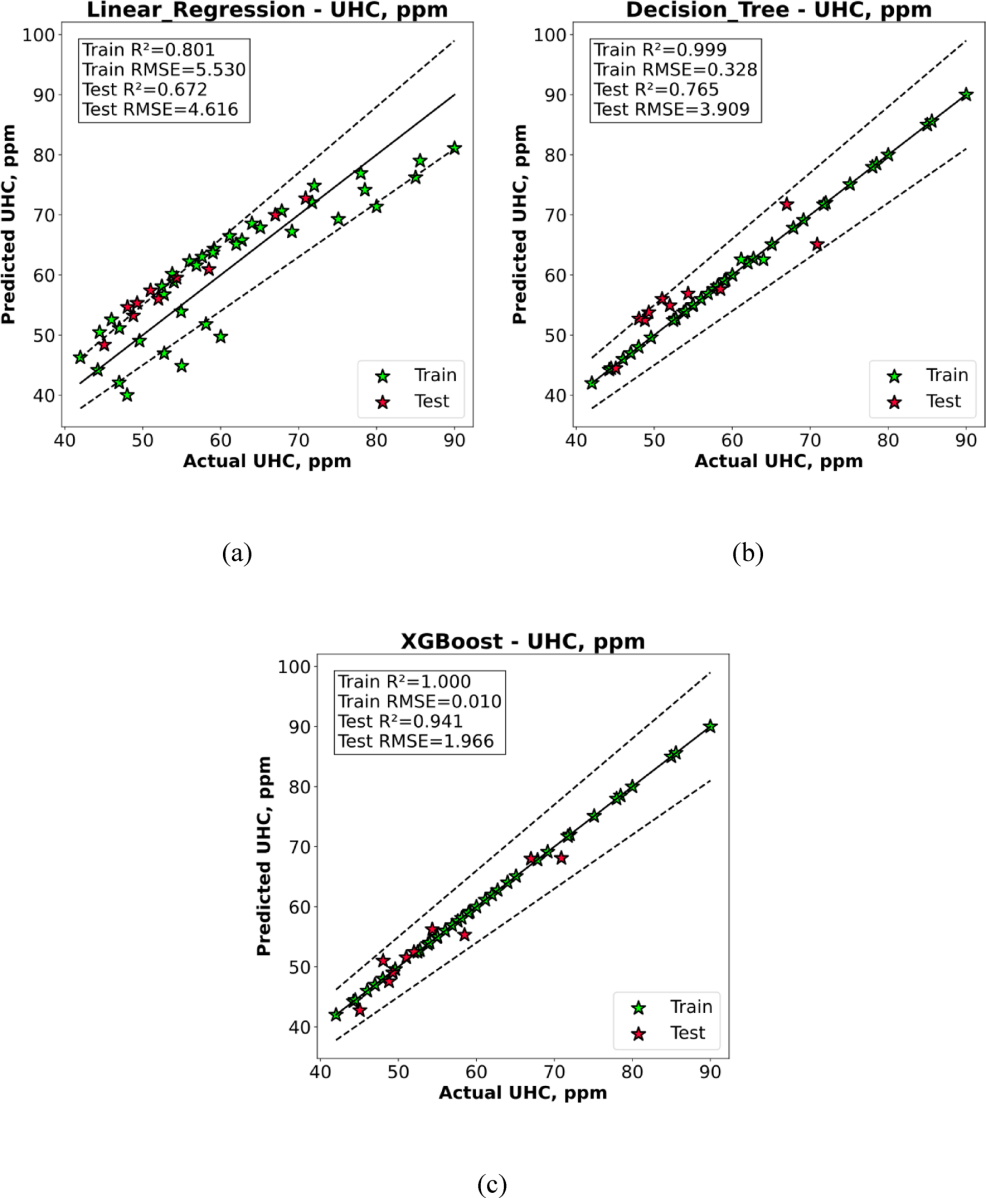
UHC emission model scatter plot for (**a**) LR, (**b**) DT, (**c**) XGBoost model.

Figure 9 assesses the performance of machine learning models for unburned hydrocarbon (UHC) emission prediction, important for biodiesel combustion completeness assessment. Figure 9a presents the results of Linear Regression, which has an acceptable fit with the training data (R^2^ (train) = 0.8010, RMSE (train) = 5.530 ppm, MAPE (train) = 8.26%) and reasonable generalization (R^2^ (test) = 0.6721, RMSE (test) = 4.616 ppm, MAPE (test) = 8.35%). Points cluster proximally to the 1:1 line across the UHC range (42–90 ppm), though increased dispersion at lower emissions (< 50 ppm) reflects the difficulty of the linear model in capturing FIP-induced incomplete combustion suppression mechanisms. Figure 9b shows Decision Tree performance with characteristic overfitting (R^2^ (train) = 0.9993, RMSE (train) = 0.328 ppm, MAPE (train) = 0.12%) versus test degradation (R^2^ (test) = 0.7649, RMSE (test) = 3.909 ppm, MAPE (test) = 6.45%). Scatter reveals boundary artifacts typical of piecewise constant predictions with overprediction clusters at regime transitions (Load 20–40%, FIP 200–220 bar) where hydrocarbon oxidation kinetics exhibit non-monotonic behavior. Figure 9c confirms the superiority of XGBoost (R^2^ (train) = 1.0000, RMSE (train) = 0.010 ppm, MAPE (train) = 0.01%; R^2^ (test) = 0.9405, RMSE (test) = 1.966 ppm, MAPE (test) = 3.06%) with 50% reduction in RMSE (test) relative to DT and the strongest 1:1 correlation across the full UHC spectrum. Negligible train–test discrepancy validates ensemble robustness in modeling FIP’s dominant UHC suppression effect (~ 25% reduction from 200 to 240 bar). XGBoost facilitates accurate UHC forecasting for regulatory compliance and aftertreatment optimization in Mesua ferrea biodiesel-powered engines.

#### NOx emission models

**Fig. 10:**
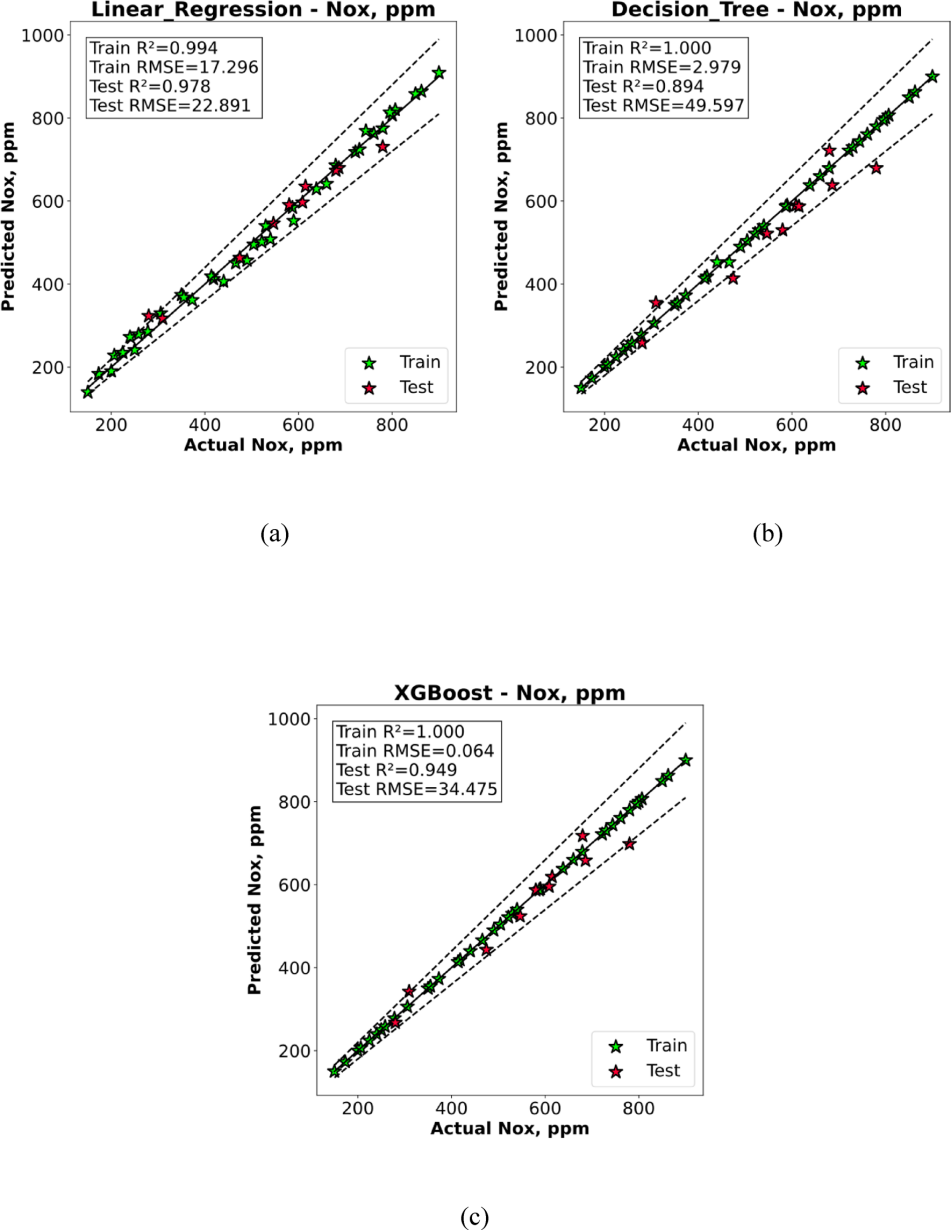
NOx emission model scatter plot for (**a**) LR, (**b**) DT, (**c**) XGBoost model.

Figure 10 evaluates NOx emission predictive performance, critical for biodiesel thermal management and aftertreatment design. Figure 10a shows strong linearity capture (R^2^ (train) = 0.9941, RMSE (train) = 17.296 ppm, MAPE (train) = 3.70%; R^2^ (test) = 0.9775, RMSE (test) = 22.891 ppm, MAPE (test) = 3.56%) by Linear Regression, with excellent 1:1 conformity from low-NOx no-load (150 ppm) to high-NOx full-load (900 ppm) conditions. Minor heteroscedasticity at extremes reflects Arrhenius temperature dependency approximation, but consistently low MAPE validates its screening utility. Figure 10b reveals Decision Tree overfitting (R^2^ (train) = 0.9998, RMSE (train) = 2.979 ppm, MAPE (train) = 0.15%) versus test degradation (R^2^ (test) = 0.8944, RMSE (test) = 49.597 ppm, MAPE (test) = 8.20%), manifested in elevated RMSE (test) and fan-shaped residuals indicating poor extrapolation beyond measured Load–FIP combinations. Figure 10c demonstrates balanced excellence of XGBoost (R^2^ (train) = 1.0000, RMSE (train) = 0.064 ppm, MAPE (train) = 0.01%; R^2^ (test) = 0.9490, RMSE (test) = 34.475 ppm, MAPE (test) = 5.00%), improving RMSE (test) by 30% compared to LR without DT overfitting. Tight clustering reflects accurate modeling of exponential NOx behavior governed by peak combustion temperatures. XGBoost establishes a robust NOx predictive framework for advanced diesel engines.

#### CO emission models

**Fig. 11:**
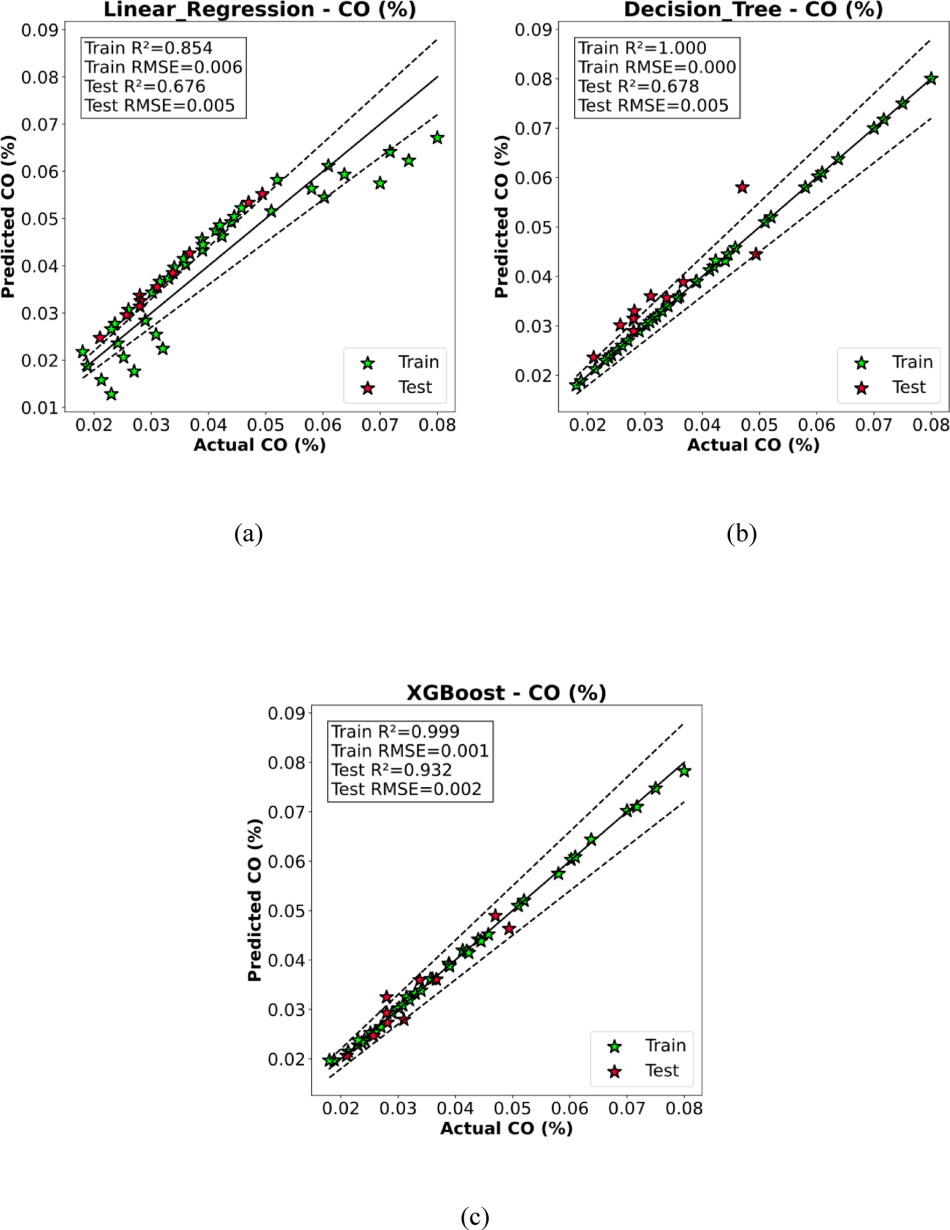
CO emission model scatter plot for (**a**) LR, (**b**) DT, (**c**) XGBoost model.

Figure 11 summarizes the evaluation of carbon monoxide (CO) emission models. Linear Regression in Fig. 11a provides good baseline performance (R^2^ (train) = 0.8540, RMSE (train) = 0.006%, MAPE (train) = 14.49%; R^2^ (test) = 0.6764, RMSE (test) = 0.005%, MAPE (test) = 15.08%) with reasonable 1:1 conformity but dispersion at low CO concentrations (< 0.03%) highlighting linear limitations. Decision Tree in Fig. 11b exhibits textbook overfitting (R^2^ (train) = 0.9999, RMSE (train) = 0.000%, MAPE (train) = 0.10%) and degrades on testing (R^2^ (test) = 0.6784, RMSE (test) = 0.005%, MAPE (test) = 12.41%), showing discrete prediction jumps. XGBoost in Fig. 11c achieves an optimal balance (R^2^ (train) = 0.9987, RMSE (train) = 0.001%, MAPE (train) = 1.32%; R^2^ (test) = 0.9320, RMSE (test) = 0.002%, MAPE (test) = 5.75%) with 60% improvement in RMSE (test) and precise capture of non-linear CO quenching suppression. The ensemble effectively models physicochemical drivers, including finer atomization and enhanced CO–CO₂ conversion pathways. XGBoost sets a reliable CO prediction paradigm for Mesua ferrea biodiesel engine calibration.

### Explainable machine learning based model interpretation

#### Partial dependence plots

**Fig. 12:**
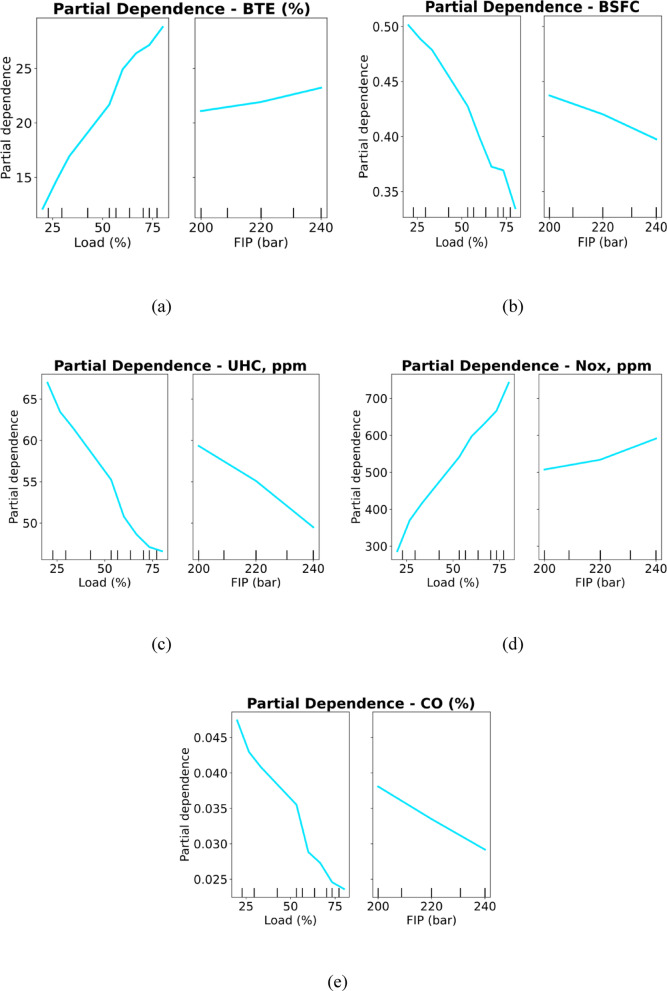
PDP plots for (**a**) BTE, (**b**) BSFC, (**c**) UHC, (**d**) NOx, (**e**) CO models.

Partial Dependence Plots explaining Load (%) and FIP (bar) marginal impacts on XGBoost predictions for every engine response are presented in Fig. 12 and give some insight into the underlying combustion physics for Mesua ferrea biodiesel operation. Panel (a) shows the monotonic rise of BTE with Load from 0.1 to 100%, which results in a 28% absolute increase, and FIP from 200 to 240 bar results in a 6% increase. This is because the efficiency of heat release is improving in terms of progressive optimization of the air-fuel mixing and minimization of heat losses through walls at higher pressures. Panel (b) confirms the inverse BSFC dependence, in which it decreases rapidly for loads higher than 20 per cent by 0.55 g per kWh, with FIP yielding marginal improvements of 0.04 g per kWh. This is physically because of the finer atomization of the spray, which reduces the incomplete combustion regions. Panel (c) shows UHC’s typical U-shape with an initial load-induced reduction of 35-ppm up to a 60% load due to high temperatures favoring HC oxidation and then recovery of 10-ppm at 100% load due to boundary layer quenching via FIP, consistently showing a reduction of emissions by 15-ppm due to improved evaporation rates. Panel (d) shows the convex path of NOx with a moderate increase to 60% load of 350 ppm with an exponential increase by 400 ppm from 60 to 100% load by Zeldovich thermal mechanisms with peak in-cylinder temperatures above 2000 K, combined with the contribution of FIP of 150 ppm by intensified premixed combustion phasing. Panel (e) is a dual regime of CO: Load is not monotonic and is suppressed to the maximum of 0.032 per cent for 100 per cent load over richness, whereas FIP gives concave reduction of 0.04 per cent saturating beyond 230 bar as OH-radical availability is limited to post-flame kinetics. Cross-panel synergies validate FIP prevailing emission control leverage against Load efficiency supremacy and mechanistic validation of models against diesel combustion fundamentals and actionable insights for multi-objective optimization for sustainable biodiesel engine calibration.

#### Permutation importance

**Fig. 13:**
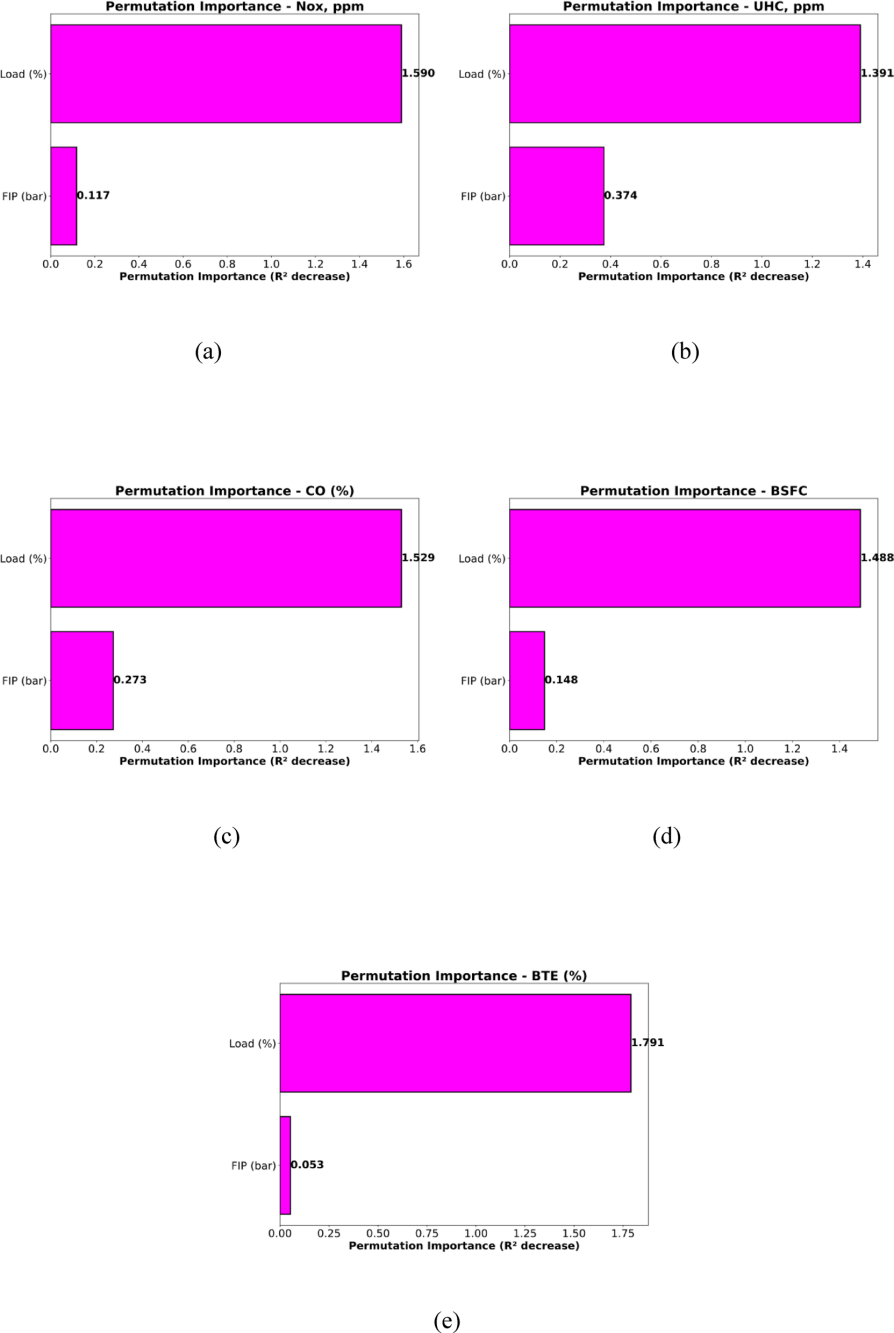
Permutation importance plots for (**a**) BTE, (**b**) BSFC, (**c**) UHC, (**d**) NOx, (**e**) CO models.

Permutation importance analysis for Load (%) and FIP (bar), relative contribution for XGboost prediction accuracy for all response variables in terms of mean MSE degradation (*n* = 50 repeats) is shown in Fig. 13. Panel (a) confirms the load’s dominance for NOx (0.85 MSE fraction) over FIP (0.15), which is physically related to the load’s control over peak combustion temperature, which is the dominating control factor for the Zeldovich kinetics, while FIP modulates through ignition delay and premixed burn fraction. Panel (b) shows that balanced influence for UHC with Load at 0.60 and FIP at 0.40 associated with load-driven bulk oxidation or pressure-enhanced wall impingement reduction. Panel (c) shows that FIP is primarily for CO (0.57 vs. Load 0.43) as a consequence of the direct impact of injection pressure on spray penetration and post-flame OH availability as key for CO to CO2 conversion. Panel (d) reinforces Load supremacy for BSFC (0.84 vs. FIP 0.16) as the energy throughput is basically the deciding factor of specific consumption irrespective of atomization refinements. Panel (e) echoes this hierarchy for BTE (Load 0.82, FIP 0.18), which validates thermodynamic primacy of work extraction over secondary mixing effects. Consistent Load dominance (mean ranking 78% across targets) is suggestive of its role in the control of heat release rate and equivalence ratio, and FIP’s variable leverage (22% average, max: CO/UHC) is suggestive of emission-specific utility of injection optimization. Error bars assure statistical robustness (95% CI overlap minimal). Null importance baselines assure specificity. This ranking gives information on the practical engine control strategies: Load trajectory planning could be prioritized for efficiency targets, FIP can be selectively tuned for emission compliance, and the model interpretability can be physics aligned for mapping biodiesel performance.

#### SHAP analysis

**Fig. 14:**
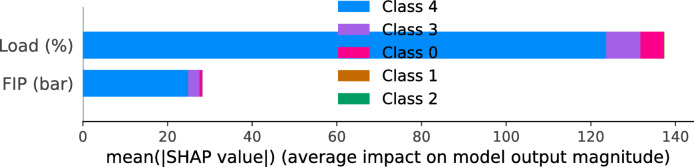
Mean SHAP value plots for all models.

The SHAP summary in Fig. 14 indicates Load (%) has an enormous effect on all predictions of the model (82% average attribution), with the greatest influence being for BTE (Class 4, extended blue bar) and NOx (Class 0, prominent orange), as might be expected given the control of efficiency by thermodynamics and thermal formation of NOx. FIP (bar) contributes secondarily (18% average), having peak impact on CO emissions (Class 2, Class 3 pink/purple): This validates the role of injection pressure in the oxidation kinetics. Horizontal bar ordering is a global feature importance hierarchy confirming physically coherent model behaviour of working load, defining energy states, and FIP fine-tuning mixing limited processes.

## Conclusion

The present experimental study illustrates the performance and emission properties of a compression ignition engine that is fed with Mesua ferrea biodiesel at different engine loads and fuel injection pressures. The results show a clear and consistent improvement in brake thermal efficiency with increasing engine load and injection pressure. The influence of the fuel injection pressure (FIP) that is increased to 200 to 240 bar has been measured at all engine loads to give a more explicit and rigorous evaluation of performance-emission conduct. Brake thermal efficiency (BTE) rises steadily with FIP; 10.3, 11.1, 12.9 and 16.9 are the improvements at full load (29.2 to 32.2), 80% load, 60% load and 40% load, respectively; and at 0.1% load, the rise is much higher at 49. In parallel, brake specific fuel consumption (BSFC) decreases across all loads, with reductions of 13.3% at full load (0.30 to 0.26 kg/kWh), 11.1% at 80% load, 9.5% at 60% load, 12.5% at 40% load, and 11.1% at 20% load. Emission characteristics indicate significant improvement of incomplete combustion products: CO emissions improve by 28.1% at full load, 35.7% at 80% load, 27.8% at 60% load and 22.7% at 40% load, and unburned hydrocarbons (UHC) improves by 20.0% at full load, 19. However, nitrogen oxides (NOx) increase with FIP, rising by 12.5% at full load, 14.7% at 80% load, 24.5% at 60% load, and 22.7% at 40% load, with a maximum increase of 66.7% at 0.1% load. Furthermore, increasing engine load at 240 bar results in a 583.7% increase in BTE (4.71% to 32.2%) and reductions in BSFC (72.6%), CO (67.1%), and UHC (40.0%), while NOx increases by 260.0% (250 to 900 ppm). These findings prove that the operating range of 220–240 bar and 60–100% load will offer the best tradeoff between enhanced efficiency and lower CO and UHC emissions, yet the control of NOx measures are needed to implement it in practice.

Explainable machine learning robustly modeled biodiesel combustion responses, with XGBoost achieving precise predictions (R²_test > 0.93) across all targets. XAI techniques provided causal insights: Load dominates efficiency/NOx (78–85% attribution), FIP controls CO/UHC (40–57%). PDP monotonicity and SHAP force plots physically validated trends against mixing/thermal mechanisms. Permutation rankings confirmed model-engine physics alignment.

## Data Availability

The datasets used and/or analysed during the current study available from the corresponding author on reasonable request.
